# Stathmin-dependent molecular targeting therapy for malignant tumor: the latest 5 years’ discoveries and developments

**DOI:** 10.1186/s12967-016-1000-z

**Published:** 2016-09-27

**Authors:** Rong Biaoxue, Cai Xiguang, Liu Hua, Yang Shuanying

**Affiliations:** 1 Department of Respiratory Medicine, First Affiliated Hospital, Xi’an Medical University, Xi’an, China; 2 Department of Respiratory Medicine, Gansu Provincial Hospital, Lanzhou, China; 3 Department of Respiratory Medicine, Second Affiliated Hospital, Xi’an Jiaotong University, Xi’an, China

**Keywords:** Cancer, Stathmin, Drug target, Anticancer therapy, Molecular targeted therapy

## Abstract

Knowledge of the molecular mechanisms on malignant tumors is very critical for the development of new treatment strategies like molecularly targeted therapies. In last 5 years, many investigations suggest that stathmin is over-expressed in a variety of human malignant tumors, and potentially promotes the occurrence and development of tumors. Rather, down-regulation of stathmin can reduce cell proliferation, motility and metastasis and induce apoptosis of malignant tumors. Thus, a stathmin antagonist, such as a specific inhibitor (antibody, small molecule compound, peptide, or siRNA), may be a novel strategy of molecular targeted therapy. This review summarizes the research progress of recent 5 years on the role of stathmin in tumorigenesis, the molecular mechanisms and development of anti-stathmin treatment, which suggest that continued investigations into the function of stathmin in the tumorigenesis could lead to more rationally designed therapeutics targeting stathmin for treating human malignant tumors.

## Background

With the development of tumor molecular biology, progress of the detection and treatment of cancer has led to an impressive reduction in both mortality and morbidity. However, cancer still remains one of the most clinically challenging disease [1]. And the current first-line chemotherapy options, such as the combination of platinum-based agents with paclitaxel, gemcitabine, vinorelbine, or docetaxel, seems to have reached a plateau of efficacy [2]. Especially, the resistance to traditional chemotherapeutic agents of tumors has become a very challenging problem. Therefore, more knowledge of the signal events of oncogenesis is required for the development of new drugs [1].

Stathmin (also known as Op18, p18, p19, stathmin 1 or metablastin) has been found to be up-regulated in some cancers [3–6] and correlates with cell differentiation, proliferation and migration, especially in solid tumor cells [7, 8]. Thus, stathmin may be an attractive target for drug design as targeting this molecule could simultaneously inhibit several aspects of tumor progression. Five years ago, Barbara Belletti [5] and Shushan Rana [6] made a summing up of relationship between stathmin and cancer. However, recent 5 years, many other studies referring the expression, mechanism and signal pathways of stathmin in tumors have been continuously reported, and some of them are very marked and impressive. It seems that this is an urgent request for cardinal significance that reviews the latest 5 years reports on new progress of this field. Here, we recapitulate the multiple roles of stathmin in cancer progression, the mechanisms and signal pathways of regulating the proliferation, apoptosis, migration of tumor cells, the pre-clinical results of stathmin inhibition in various cancer models, and available data as rationale for the therapeutic manipulation of stathmin in cancer patients.

### Stathmin: molecular structure and function domain

**Fig. 1:**
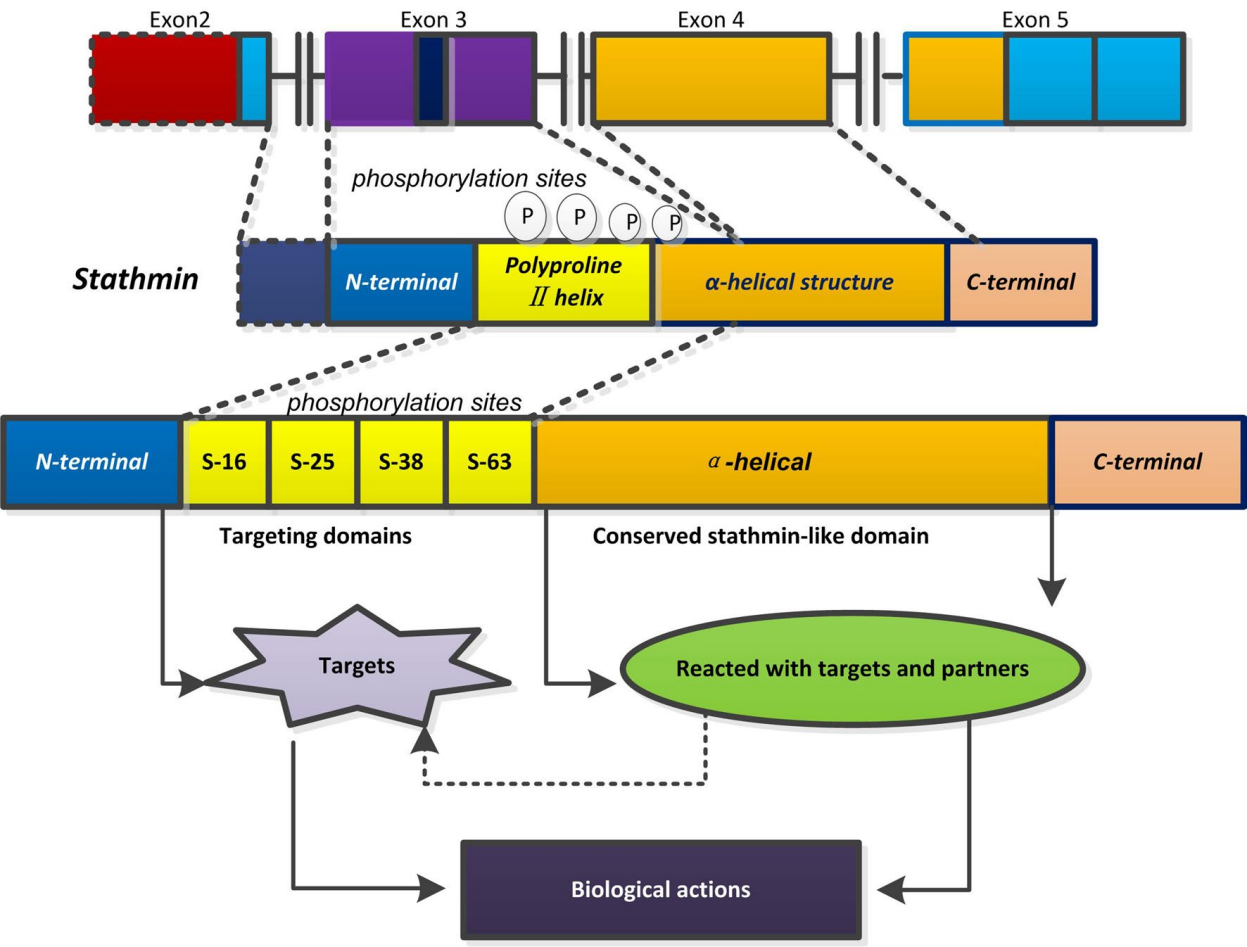
Structure diagram of stathmin and related signal patterns. Stathmin has a highly conserved stathmin-like domain (α-helical structure) and has four positions of serine phosphorylation sites (**S16, S25, S38 and S63**). The N- and C-terminal of stathmin exert different functions when stathmin participate the molecular actions. Some downstream target and/or partner proteins participate the modulation process of stathmin and interact each other to exert biological actions through phosphorylation of stathmin

In spite of many years of research, knowledge of this molecule is still so obscure that some investigators even did not define its name certainly, which dues to the fact that it is identified independently in many different research institutes. So, it is called by different names (e.g., p17, p18, p19, 19 K, metablastin, oncoprotein 18, LAP18, and stathmin 1, Op18/stathmin). Stathmin is composed of 149 amino acids, which are organized into four domains (I–IV), and the core region (amino acids 42–126) is site for tubulin interaction with the additional requirement of either an N- or C-terminal extension [9]. The members of stathmin family belong to microtubule-regulating proteins, which include stathmin (stathmin 1), SCG10, SCLIP, and RB3/RB3′/RB3′’coded by four different genes [9]. And, all stathmin family members have a highly conserved stathmin-like domain. The stathmin-like domains of these proteins also possess a tubulin binding activity (Fig. 1).

The N-terminal and C-terminal of stathmin exert different functions when stathmin participate the molecular actions. The N-terminal of stathmin is the regulatory domain of stathmin. There are four phosphorylation domains on this region, designated as Ser 16, 25, 38 and 63 [5], which intimately correlate with the functions of stathmin by kinases involved in major intracellular regulatory cascades. The C-terminal is interaction domain of stathmin, which includes a coiled-coil forming a-helical structure, potentially interacting with other different signal proteins to exert biological actions [10]. Stathmin is an ubiquitous cytosolic phosphoprotein, proposes to be a small regulatory protein and a relay integrating diverse intracellular signaling pathways involved in the control of cell proliferation, differentiation and activities [5]. Some downstream target and/or partner proteins participate the modulation process of stathmin and interact each other to exert biological actions through phosphorylation of stathmin (Fig. 1b).

### Expression of stathmin in human malignant tumor

Stathmin expression has been examined in several types of cancer. We emphatically harvested the lasted 5 year studies on this field. As shown in Table 1, stathmin is highly expressed in a variety of assessed human malignancies including lung cancer [3, 11], esophageal carcinoma [12–16], breast cancer [17], cholangiocarcinoma [18], hepatocellular carcinoma [4, 19, 20], gastric cancer [21–25], pancreatic cancer [26–28], myelodysplastic syndromes [29], nasopharyngeal carcinoma [30], malignant pleural mesothelioma [31], cervical carcinomas [32], endometrial carcinoma [33, 34], urothelial carcinoma of the bladder [35–37], colorectal cancer [38] and glioblastoma [39]. And, high expression of stathmin intimately correlates with the malignant behavior and clinical features of tumor.

**Table 1:** Summary of stathmin expression in human tumors and correlation with clinical outcome

Publications	Cancer type	Cell lines	Tissue	Techniques	Notes
Nie [3]	Lung cancer	Yes	Yes	qRT-PCR, IHC	Overexpression of stathmin is a poor prognostic biomarker for non-small cell lung cancer
Sun [11]		No	Yes	IHC, WB, qRT-PCR	Overexpression of stathmin correlates with shorter overall survival and progression-free survival in non-small cell lung cancer
Wang [15]	Esophageal carcinoma	No	Yes	IHC, ISH	Stathmin is associated with esophageal carcinoma (EC) development and progression and may be a good prognostic marker for patients with EC
Wang [16]		Yes	Yes	IHC, WB	Stathmin is highly expressed in esophageal squamous cell carcinoma Eca109 and TE-1 cells
Liu [13]		Yes	Yes	2-DE and IHC	Stathmin is overexpressed in esophageal squamous cell carcinoma (ESCC) tissues
Akhtar [12]		No	Yes	IHC, WB	Stathmin overexpression predicts a high risk for lymphatic metastatic recurrence in pN0 esophageal squamous cell carcinoma patients
Baquero [17]	Breast cancer	No	Yes	IHC	High stathmin expression predicts worse overall survival of breast cancer
Watanabe [18]	Cholangiocarcinoma	Yes	Yes	IHC, WB	Stathmin correlates with shorter recurrence-free survival and overall survival in cholangiocarcinoma patients
Hsieh [4]	Hepatocellular carcinoma	Yes	Yes	IHC, WB	Stathmin overexpression in hepatoma is associated with local invasion, early recurrence, and poor prognosis, and is an independent indicator for tumor recurrence
Ahn [19]		Yes	Yes	IHC, WB	Stathmin and EF1α increase as multistep hepatocarcinogenesis progressed, showing the highest levels in hepatocellular carcinomas
Chen [20]		Yes	Yes	IHC, WB	Upregulation of E2F1 and stathmin are associated with worse outcomes in patients with hepatocellular carcinoma
Li [24]	Gastric cancer	Yes	Yes	IHC, WB	Stathmin is overexpressed in 103 post-operational gastric cancer specimens
Liu [25]		Yes	Yes	IHC, WB	Stathmin is elevated in gastric cancer tissues, indicating a possible association between the stathmin and the disease occurrence
Batsaikhan [21]		No	Yes	IHC, ISH	Higher stathmin is significantly associated with gender-and poorly differentiated gastric adenocarcinoma
Kang [22]		Yes	Yes	IHC,WB, qRT-PCR	Stathmin is upregulated in gastric cancer cell lines and primary gastric adenocarcinomas, which is correlated with age, T stage and lymph node metastasis
Ke [23]		No	Yes	IHC, WB, qRT-PCR	Stathmin mRNA and protein in gastric cancer tissues are overexpressed, which correlates with Lauren’s classification, depth of invasion, lymph node metastases, and tumor node metastasis (TNM) stage
Lu [27]	Pancreatic cancer	Yes	Yes	IHC, WB	Stathmin is over-expressed in pancreatic cancer tissues and correlates with vascular emboli, tumor size, and overall survival
Schimmack [28]	Pancreatic neuroendocrine neoplasm	Yes	Yes	IHC,WB, qRT-PCR	Stathmin mRNA and protein are overexpressed in pancreatic neuroendocrine neoplasm (pNENs) and correlate with pNEN tumor extension, size, and Ki67 expression
Li [59]		Yes	Yes	IHC, WB	Stathmin is overexpressed to a large extent in pancreatic cancer tissues and cell lines
Machado-Neto [29]	Myelodysplastic syndromes	Yes	Yes	IHC, WB	Higher stathmin level is observed in proliferating hematopoietic cells, high-risk myelodysplastic syndromes (MDS) and acute leukemia cells
Hsu [30]	Nasopharyngeal carcinoma	Yes	Yes	IHC, WB	Higher stathmin expression is correlated with advanced age higher T stage and overall clinical stage
Birnie [31]	Malignant pleural mesothelioma	Yes	No	IHC, WB	Stathmin expression is higher in malignant pleural mesothelioma cell lines when compared with primary mesothelial cell controls
Howitt [32]	Cervical carcinomas	No	Yes	IHC	Stathmin is overexpressed in virtually all cervical carcinomas and cervical intraepithelial neoplasias 3 (CIN3) lesions
He [33]	Endometrial carcinoma	Yes	Yes	IHC	Stathmin is up-regulated in endometrial carcinoma (EC), and elevated stathmin is correlated positively with tumor stage and lymph node metastasis
Wik [34]		Yes	Yes	IHC, FISH, FCM, SNP	High p-stathmin(S38) level is associated with poor prognosis, independent of other features.
Bhagirath [37]	Bladder urothelial carcinoma	No	No	ELISA, qRT-PCR	The urinary level of serum stathmin concentration shows a specific increase in patients with urothelial carcinoma of the bladder as compared to the controls
Wosnitzer [35]		No	Yes	Immunophenotype analysis	Increased total tau (cytoplasmic and nuclear) and stathmin before intravesical taxane therapy is significantly associated with decreased recurrence-free survival
Hemdan [36]		No	Yes	IHC,WB	High stathmin expression correlates to shorter disease-specific survival hazard ratio, elevated p53 and Ki67-protein levels
Tan [38]	Colorectal cancer	Yes	Yes	2-D DIGE	Stathmin is found to be highly up-regulated in colorectal cancer E1 cells as compared to HCT-116 cells
Marie [39]	Glioblastoma	Yes	Yes	qRT-PCR	Stathmin expression is significantly increased in malignant diffusely infiltrative astrocytomas compared with pilocytic astrocytoma

*qRT-PCR* real-time quantitative reverse transcription polymerase chain reaction, *IHC* immunohistochemistry, *WB* west blotting, *ISH* in situ hybridization, *2-DE* two-dimensional gel electrophoresis, *FISH* fluorescence in situ hybridization, *FCM* flow cytometry, *SNP* single nucleotide polymorphism, *ELISA* enzyme-linked immunosorbent assay, *2-D DIGE* two dimension difference gel electrophoresis, *EC* esophageal carcinoma, *ESCC* esophageal squamous cell carcinoma, *EF1α EF1α promoter*, E2F1 E2F transcription factor 1, *mRNA* messenger RNA, *TNM* tumor node metastasis classification of malignant tumours, *pNENs* pancreatic neuroendocrine neoplasm, *Ki67* Ki67 gene, *MDS* myelodysplastic syndromes, *CIN3* cervical intraepithelial neoplasias 3 grades, *EC* endometrial carcinoma, *E1 and HCT-116* colorectal cancer cell lines

With regard to detection of stathmin, most of the studies adopted two levels of cell and tissues. immunohistochemistry (IHC) analysis for stathmin was adopted by almost every study and some even include west blotting. However, most if not all of the studies are concordant with the notion that stathmin expression and/or activity are up-regulated in human cancer. Specifically, seven studies adopted extra detection of qRT-PCR [3, 11, 22, 23, 28, 37, 39] and two adopted the methods of 2-DE [13, 38], which are all belonged to available methods. Impressively, stathmin overexpression is associated with poor survival and local or distant metastasis formation in several types of human cancer including lung cancer [11], esophageal carcinoma [12], breast cancer [17], cholangiocarcinoma [18], hepatocellular carcinoma [4, 20], gastric cancer [22, 23, 25], pancreatic cancer [27] and endometrial carcinoma [33–35, 37]. These data strongly indicate that stathmin has a potential to become a prognostic index and tumor marker for malignant cancers.

However, there are some limitations on these studies. First, only surgical specimens were used in some studies, which results in a major patient selection bias. Second, the number of samples of some studies is relatively small. Thus, further investigations into different cancers extensively involving a larger number of samples of patients is required to reach a more definitive conclusion. Despite all this, these studies elucidate that stathmin potentially contributes to tumor development and progression, suggesting that its high expression is not only required, but also detrimental during stages of cancer onset [40].

### New progress regarding to biological function of stathmin on malignant tumor

#### Stathmin interferes with microtubule dynamics on cancer cells

**Fig. 2:**
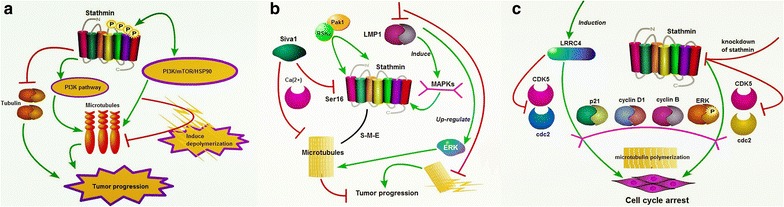
Stathmin interferes with microtubule dynamics. **a**There is a stathmin-microtubule-EMT (S-M-E) axis during cancer development; stathmin promotes malignant potential in cancer cells by initiating EMT; phosphoinositide 3-kinase (PI3 K)/mTOR/HSP90 are suggested as possible targets in p-stathmin(S38)-high cases; **b** siva1 enhances the formation of microtubules and impedes adhesion, cell migration, and EMT by inhibiting stathmin’s activity; inhibition of LMP1 expression attenuates the interaction of ERK with stathmin and promotes microtubule depolymerization; **c** induction of LRRC4 or knockdown of stathmin induces cell cycle arrest by modulating the p21, cyclin D1, and cyclin B expression, and the ERK phosphorylation. *PI3* *K* phosphoinositide 3-kinase, *mTOR* mammalian target of rapamycin, HSP90, heat shock protein 90, *EMT* epithelial-mesenchymal transition, *Siva* proapoptotic protein, *LMP1* latent membrane protein 1, *ERK* extracellular regulated protein kinases, *RSK2* p90 ribosomal S6 kinase 2, *LRRC4* leucine rich repeat containing 4, *CDK5* cyclin-dependent kinase-5, *CDC2* cyclin-dependent kinase 1, *p21* cyclin-dependent kinases

This part mainly describes the relationship between regulation of microtubule dynamics and role of stathmin in malignant tumors, as well as prospects or proposals of research. Microtubules are essential for the structure and function of the cell that relate to intracellular transport, cell motility and polarity [6]. When cell turns into mitosis, microtubules become the major component of mitotic spindle which enables correct chromosome segregation [10]. As shown in Fig. 2a, the alteration of microtubules is tided up with many crucial processes such as cell proliferation, mitosis and motility, which is controlled by the phosphorylation of stathmin by two phases causing biphasic shifts in microtubules stability/instability [41]. Epithelial-mesenchymal transition (EMT) plays a positive role in growth and migration of tumor cells. As a microtubule-destabilizing protein, stathmin can promote malignant potential for cancer cells by initiating EMT [10]. Research shows the microtubule-destabilizing activity of stathmin contributes to EMT via stathmin-microtubule-EMT (S-M-E) axis during cancer development [10]. Phosphoinositide 3-kinase PI3 K/mTOR/HSP90 is shown as a possible signal target for p-stathmin S38- high-endometrial cancer cases. High p-stathmin (S38) correlates with PI3 K pathway and increase PIK3CA copy number (FISH) and a PI3 K activation score [34].

Siva1, an apoptosis-inducing factor, inhibits stathmin’s activity directly as well as indirectly through Ca(2+)/calmodulin-dependent protein kinase II-mediated phosphorylation of stathmin at Ser16, which enhances the formation of microtubules and impedes focal adhesion assembly and EMT. And low levels of Siva1 and Ser16-phosphorylated stathmin correlate with high metastatic states of human breast cancer cells [42]. The p90 ribosomal S6 kinase 2 (RSK2) has been identified to promote tumor metastasis; latest one study demonstrates that RSK2 directly phosphorylates stathmin and regulates microtubule polymerization to provide a pro-invasive and pro-metastatic advantage to cancer cells [43].

One potential target of the MAPKs is stathmin and the activity of MAPK is induced by the Epstein-Barr virus-encoded latent membrane protein 1 (LMP1). Research shows LMP1 regulates stathmin signaling, which is mainly mediated by ERK. The inhibition of LMP1 expression attenuates the interaction of ERK with stathmin and promotes microtubule depolymerization [44]. Stathmin depletion causes significant inhibition of HGF-induced WAVE2 transport and lamellipodia formation. Pak1 plays a critical role in this effect on phosphorylation and recruitment of tubulin-bound stathmin/Op18 to the complex [45]. Stathmin silencing also reduces the activity of CDC25, Aurora A and Plk1. Research shows MTs contribute to Plk1 activation, and stathmin regulates mitotic entry via MTs to control localization and activation of both Aurora A and Plk1 [46] (Fig. 2b).

In the interphase of cell cycle, increased stathmin damages nucleation from centrosome. Homo sapiens leucine rich repeat containing 4 (LRRC4) is epigenetically inactivated commonly in glioma. Knockdown of stathmin induces cell cycle arrest of glioma U251 cells and increases the microtubulin polymerization of U251 cells. And down-regulation of stathmin inhibits CDK5 and cdc2 kinase, which correlates with the modulation of the p21, cyclin D1, and cyclin B expression, and the situation of ERK phosphorylation [47] (Fig. 2c). Overall, the microtubule-destabilizing protein stathmin is involved in cancer development. Most interestingly, stathmin and its microtubule-depolymerizing activity intimately correlate with EMT progress, which is involved in tumor malignant progression and recurrence, even in resistance of chemotherapy. Thus, further investigations into this process of stathmin-microtubule dynamics should be done.

#### Stathmin correlates with proliferation of cancer cells

**Fig. 3:**
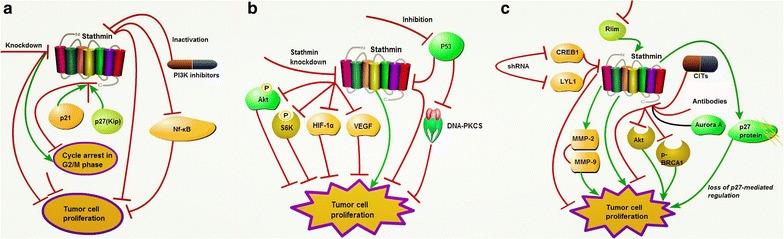
Stathmin affects the proliferation of cancer cells. **a** Stathmin cooperates with p21(Cip1/Waf1) and p27(Kip) to control the early phase of G1 to S phase and reduces tumor growth by down-regulation of Nf-κB; **b** stathmin knockdown inhibits the expression of HIF-1α and VEGF and phosphorylation of S6 K and Akt; stathmin binds phosphorylation of p53(MUT) by DNA-PKCS, but inhibition of stathmin or DNA-PKCS results in M phase failure by impairing p53(MUT)-dependent transcription; **c** down-regulation of CREB1 and LYL1 reduces cell proliferation by the down-regulating stathmin; knockdown of stathmin promotes the effects of indoly-chalcones CITs; stathmin enhances growth and invasion of EC by activating MMP2 and MMP9; inhibition of Rlim increases expression of stathmin and leads to cell proliferation; inhibition of Aurora A by stathmin promoter inhibits cells proliferation by reducing expressions of phosphatidylinositol 3 kinase/Akt and p-BRCA1; stathmin potentiates cell proliferation by regulating function of p27. *p21* cyclin-dependent kinases, *p27* cell cycle checkpoints regulator protein, *PI3* *K* phosphoinositide 3-kinase, *Nf-κB* nuclear factor ‘kappa-light-chain-enhancer’ of activated B-cells, *Akt* v-akt murine thymoma viral oncogene, *S6* *K* ribosomal protein S6 kinase 1, *HIF-1α* hypoxia-inducible factor-1, *VEGF* vascular endothelial growth factor, *p53* tumor suppressor p53, *DNA-PKCS* catalytic subunit of the DNA-dependent protein kinase, *CREB1* leucine zipper transcription factor, *LYL1* basic helix-loop-helix transcription factor, *Rlim* a Ring H2 zinc finger protein, *MMP* matrix metalloproteinases, *p-BRCA1* phosphorylated phosphorylated breast cancer gene 1, *CITs*, indoly-chalcones

Stathmin is an important member of a family of microtubule-destabilizing proteins [48], which has been proved to exert critical actions in control of cell proliferation [5]. Several observations suggest that there is a close link between stathmin expression and/or phosphorylation and regulation of cellular proliferation in cancers. Knockdown of stathmin leads to cell cycle arrest in G2/M phase in esophageal carcinoma cells [16] and pancreatic cancer cells and clonogenicity of Namalwa leukemia cells [29], and reduces the viability and colony formation. Adenovirus-mediated gene transfer of anti-stathmin ribozyme results in a dose-dependent inhibition of proliferation and clonogenicity associated with a G2/M arrest and increases the apoptosis rate of both ER-positive and ER-negative breast cancer cells [48]. The CDK inhibitor p27(kip1) and p21(Cip1/Waf1) are critical regulators of cell cycle progression, which bind to stathmin as partners to control the early phase of G1 to S phase transition to the context of tumor progression [49]. Knockdown of stathmin results in a decrease in cellular proliferation and invasion in lung cancer cells [3] and in pancreatic neuroendocrine neoplasm cells, and PI3 K inhibitors directly inhibits proliferation via stathmin inactivation [28]. Furthermore, silence of stathmin down-regulates the expression of Nf-κB (p65), which indicates that stathmin might play its oncogenic role by an interaction with Nf-κB pathway [27] (Fig. 3a).

Stathmin depletion suppresses the expression of hypoxia-induced factor-1α (HIF-1α) and VEGF, and impedes the phosphorylation of ribosomal protein S6 kinase 1 (S6 K) and Akt, which means stathmin play a critical role in the mTOR/HIF-1α/VEGF signally pathway [50]. At molecular level, stathmin favours the binding and the phosphorylation of p53(MUT) by catalytic subunit of the DNA-dependent protein kinase (DNA-PK_CS_), modulating p53(MUT) stability and transcriptional activity. Inhibition of stathmin or impediment of DNA-PK_CS_ damage the p53(MUT)-dependent transcription lead to the failure of M phase and the death of epithelial ovarian carcinomas (EOC) cells [8] (Fig. 3b). The down-regulation of leucine zipper transcription factor (CREB1) and helix-loop-helix transcription factor (LYL1) reduce the expression of stathmin, which lead to inhibition of cell proliferation [51]. Knockdown of stathmin promotes the effects of indoly-chalcones CITs (CIT-026, CIT-214, CIT-223) to bring down microtubule destabilization, result in cell death and decelerate cell proliferation [52]. In addition, stathmin enhances the growth and invasion of endometrial carcinoma cells by regulating the secretion and activation of MMP2 and MMP9 [33]. And inhibition of Rlim (a Ring H2 zinc finger protein) increases the expression of stathmin, and leads to cell proliferation of human osteosarcoma cell lines [53]. Monoclonal antibodies against stathmin and paclitaxel have bee used alone or incombination to inhibit the proliferation of human lung carcinoma QG-56 cells, especially result in a significant apoptosis [54]. A novel tumor-specific RNA interference adenovirus system targeting Aurora A by using stathmin promoter not only inhibits the cells proliferation, but also enhance the chemosensitivity to paclitaxel in human breast carcinoma SK-BR-3 and MDA-MB-231 cells, and further decreases the phosphatidylinositol 3 kinase/Akt and p-BRCA1 protein expression [55]. Stathmin also plays a role in the development of fallopian tube epithelium (FTE) tumor, which potentiate aberrant cell proliferation, migration, and/or loss of polarity during early tumorigenesis, resulting from loss of p27-mediated regulation [56] (Fig. 3c).

#### Stathmin correlates with apoptosis of cancer cells

**Fig. 4:**
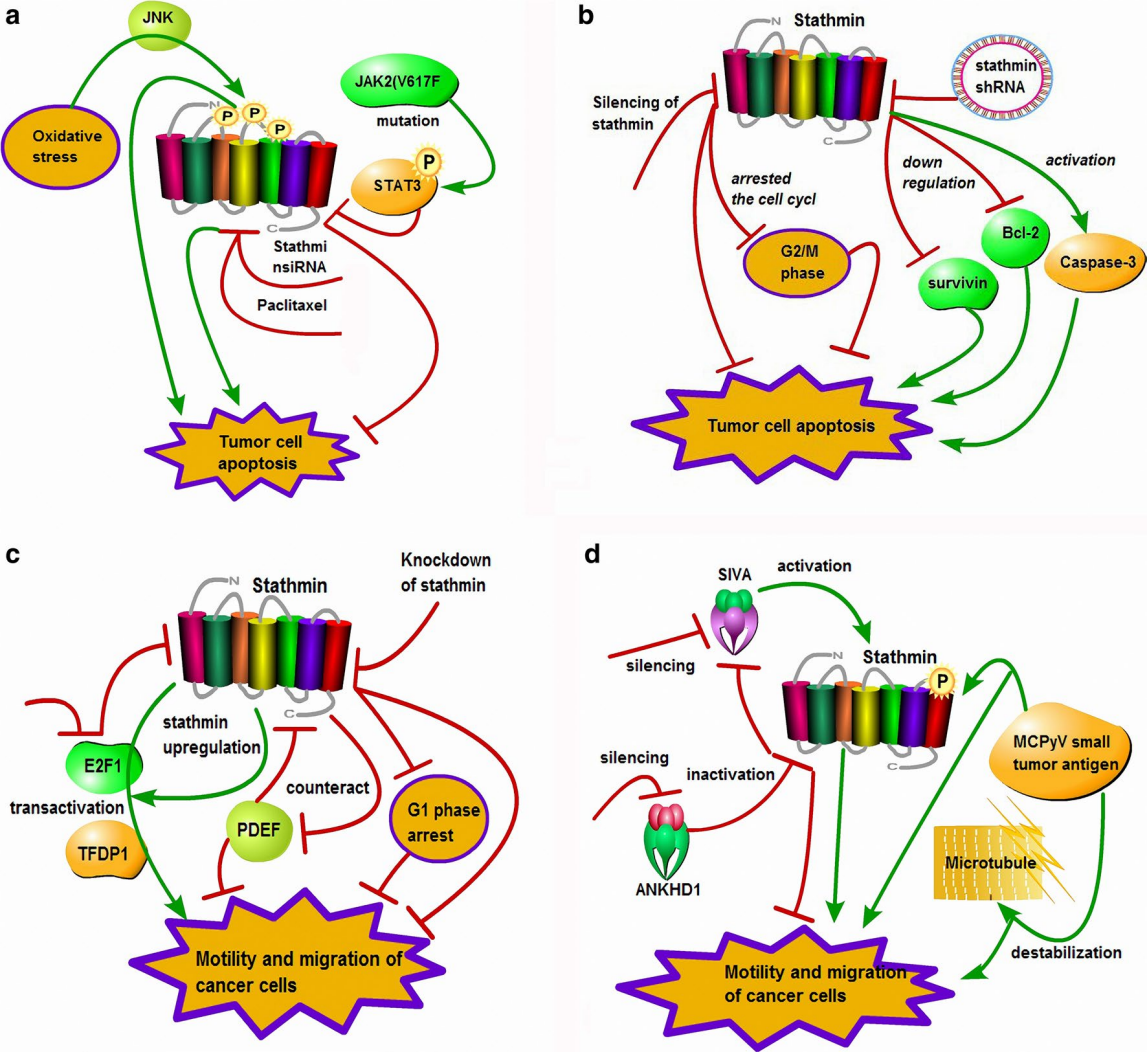
Stathmin correlates with apoptosis and mobility of cancer cells. **a** Oxidative stress induces JNK-dependent stathmin phosphorylation, but down-regulation of stathmin promotes apoptosis of cells and inhibits proliferation and migration; stathmin silencing with paclitaxel enhances tumor cell apoptosis; JAK2(V617F) mutation potentially leads to inhibition of stathmin activity via STAT3 phosphorylation; **b** silencing of stathmin increases apoptosis of cells by down-regulating Bcl-2 and survivin and activating Caspase-3, and significantly arrests the cell cycle at G2/M phase; **c** stathmin attributes to E2F1 and/or Dp-1 (TFDP1) transactivation, knockdown of the E2F1 suppresses cancer cell migration; stathmin counteracts PDEF’s effects against cell migration; **d** SIVA silencing increases cell migration by promoting stathmin activity, but ANKHD1 silencing leads to stathmin inactivation likely through inhibition of SIVA/stathmin association; MCPyV tumor antigen promotes the destabilization of the host cell microtubule network by regulating phosphorylation of stathmin, which leads to migratory cell phenotype. *JNK* c-JunN-terminalkinase, *JAK* janus kinase, *JAK* janus kinase, *STAT* signal transducers and activators of transcription, *Bcl-2* B cell lymphoma-2, *E2F1* E2F transcription factor 1, *TFDP1* transcription factor Dp-1, *PDEF* prostate-derived Ets transcription factor, *SIVA* proapoptotic protein, *ANKHD1* ankyrin repeat and KH domain containing 1 protein, *MCPyV* Merkel cell polyomavirus

Apoptosis, an orchestrated event in which cells are programmed to die after receiving specific stimuli, is an important component of cell growth control [57]. So far, many studies demonstrate that stathmin present an anti-apoptotic activity to prompt the progress of tumor cells and play an important role in control of cell cycles, which are involved in many signal molecules. Oxidative stress from menadione-generated superoxide induces JNK-dependent stathmin phosphorylation at Ser-16, Ser-25 and Ser-38 in hepatocytes. Down-regulation of stathmin promotes the sensitivity of apoptotic and necrotic cell death from menadione in hepatocytes [58]. Suppression of stathmin not only inhibits the proliferation, migration and invasion of pancreatic cancer and nasopharyngeal carcinoma cells, but also enhances the apoptosis of cancer cells [59, 60]. And, stathmin knockdown improves the chemosensitivity of gastric cancer cells to docetaxel, making the percentage of cells at the sub-G1 stage increase and promote apoptosis [61]. Research shows paclitaxel reduces the expression of stathmin, and combination of stathmin silencing with paclitaxel treatment enhances microtubules polymerization and tumor cell apoptosis [60]. A selective JAK1/2 inhibitor, ruxolitinib, has been reported to inhibit the JAK/STAT pathway in myeloproliferative neoplasms. The JAK2(V617F) mutation potentially leads to inhibition of stathmin activity via constitutive STAT3 phosphorylation. Therefore, combination of stathmin silencing and ruxolitinib treatment can reduce cell proliferation and clonal growth, and increase apoptosis induced by ruxolitinib [62] (Fig. 4a).

Glioma is common angiogenic tumor, which is always resistant to chemotherapy and radiotherapy. Research shows knockdown of stathmin inhibits the proliferation of glioma cells, induces apoptosis, arrests the cell cycle at G2/M phase in glioma stem cells (GSCs), and also suppresses the migration/invasion [63]. Down-regulation of stathmin transfected with stathmin shRNA significantly inhibits cell proliferation and tumorigenicity, arrests cell cycle in the G2/M phase and induces cell apoptosis. Furthermore, down-regulation of stathmin result in downregulation of Bcl-2 and survivin proteins, activation of Caspase-3, which all are intimately related to the development and progress of tumors [14] (Fig. 4b).

#### Stathmin expression correlates with motility of cancer cells

The mobility and migration is very critical biological event in progress and proliferation of malignant tumors. If the tumor cells become migratory, this will possibly lead to the local and remote metastasis of malignant cells, which eventually speed up the disease progress. Stathmin is important member of microtubule-regulating proteins, cell migration is intimately associated with stathmin that interfere with microtubule dynamics by elevating depolymerization of microtubules. Overexpression of stathmin is strongly related to tumor aggressiveness of nasopharyngeal carcinoma patients, which attributes to the transactivation of transcription factor 1 (E2F1) and/or transcription factor Dp-1 (TFDP1) [30]. Down-regulation of E2F1 results in reduction of stathmin in HCC lesions, suggesting that stathmin gene is transactivated by the E2F1 protein [20]. In addition, stathmin silencing significantly impedes cell proliferation and mobility of neuroblastoma cells, polyploidy of hepatoma cells [4] and esophageal squamous cell carcinoma cells [13], and remarkably retards cell migration and invasion [64]. Inversely, over-expression of stathmin enhances cell invasion and causes polyploidy of hepatoma cells [4]. When the expression of stathmin is down-regulated in gastric adenocarcinoma cells, this significantly reduces cell proliferation, colony formation and cell invasion and migration ability, and arrest cells in G1 phase [22]. It is reported that prostate-derived Ets transcription factor (PDEF) is present in breast and prostate cells and tissues. It seems that high expression of stathmin brings down the effects that PDEF inhibit cell proliferation, colony formation and tumor migration, and discloses that PDEF exert an antitumor effects through down-regulating the expression of oncogenic stathmin [65] (Fig. 4c).

It is reported that the knockdown of proapoptotic protein SIVA activates the expression of stathmin, which promotes cell mobility and migration and the growth of xenotransplanted tumors, but silencing of ankyrin repeat and KH domain containing 1 protein (ANKHD1) plays an inverse function that leads to stathmin inactivation, inhibits cell migration and the growth of xenotransplanted, which possibly depends on the inhibition mechanism of SIVA/stathmin pathway [66]. Merkel cell carcinoma (MCC) can be caused by Merkel cell polyomavirus (MCPyV), which is an aggressive skin malignant tumor, and the migratory mechanism of MCC is more likely related to the function of stathmin. It is shown that MCPyV small tumor antigen enhances microtubule destabilization of the MCC cells by modulating the phosphorylation status of stathmin, which results in the motility, migration and metastasis of Merkel cell carcinoma [67] (Fig. 4d). Although many studies have identified that there is a strong relationship between overexpression of stathmin and increased migration and/or metastatic trend of malignant tumors; much still has not been disclosed about the molecular signally pathways how stathmin performs the role on migration and metastasis in cancer cells and how stathmin interactions with those special genes and proteins.

### Stathmin and small non-coding RNAs

Recent studies have revealed that small non-protein-coding regulatory RNAs (miRNAs, MicroRNAs) may regulate complex biological processes of malignant tumors including cell proliferation, differentiation and apoptosis. So far, over 2588 miRNAs have been identified in humans and the number of investigations is growing. Evidence on the relation between MicroRNAs and malignant tumor has been suggested that some aberrant miRNA expressions promote the development of cancers, but the others play a negative function in tumorigenesis [68].

**Fig. 5:**
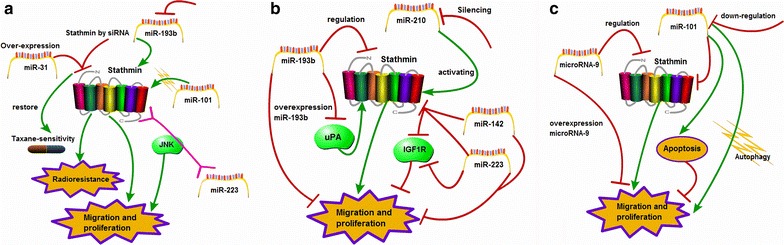
Study progress of stathmin and MicroRNAs. **a** Over-expression of miR-31 restores chemo-response by reducing stathmin expression; miR-101/stathmin pathway contributes to radioresistance in human NPC; down-regulation of miR-193b promotes migration and proliferation of tumor cells by targets stathmin; miR-223 regulates stathmin by JNK signaling pathway to regulate MPM cell motility; **b** up-regulation of miR193b reduces proliferation and migration by inhibiting stathmin and uPA; silencing of miR-210 promotes proliferation of cancerous cells; transfection of miR-142 and miR-223 decreases expression of stathmin and IGF-1R to inhibit proliferation of cancerous cells; **c** microrna-9 inhibits cell proliferation, vasculogenic mimicry and tumor growth through controlling stathmin expression; miR-101 suppresses autophagy via targets stathmin and down-regulation of miR-101 is linked to the increase of cellular proliferation and invasiveness. *miRNAs* small non-coding regulatory RNAs, *JNK* c-JunN-terminalkinase, *uPA* urokinase-type plasminogen activator, *IGF-1R* insulin-like growth factor-1 receptor, *MPM* malignant pleural mesothelioma

The chemoresistant ovarian cancer KF-TX cells present overexpression of stathmin, which is being considered to correlate with drug resistance. Down-regulation of stathmin can partly renew taxane-sensitivity of KF-TX cells, and up-regulation of miR-31 can significantly recover chemo-sensitivity of KF-TX cells (KF-TX-miR-31) by reducing stathmin expression as well [69]. Stathmin is identified as an effective functional target of miR-101, which is related to cell proliferation, radioresistance of nasopharyngeal carcinoma (NPC) cells. The miR-101 exert a critical action in radioresistance by modulating the expression of stathmin via miR-101/stathmin pathway [70]. Down-regulation of miR-193b is closely associated with overexpression of stathmin in melanoma, which is identified to be conducive to promote the migration and proliferation of tumor cells [71]. And study reveals that aberrant miR-223 contributes to aggressiveness of malignant pleural mesothelioma (MPM) by regulating stathmin and both are also in turn regulated by the JNK signally pathway [31] (Fig. 5a).

Elevation of miR-193b impedes the ability of esophageal cancer cells to recover following 5-fluorouracil (5-FU) treatment through the regulation of autophagy, which suggests that it may mediate some of its effects through stathmin regulation (potential autophagy regulator) [72]. Interestingly, overexpression of miR-193b suppresses the proliferation, migration and invasion of pancreatic cancer Panc-1 cells by inhibiting the expression of stathmin and urokinase-type plasminogen activator (uPA) [26]. It is suggested that long term colonization of Helicobacter pylori in gastric mucosa increases the risk of gastric cancer. Stathmin is considered as a target of miR-210 and down-regulation of miR-210 increases the proliferation of gastric epithelial cells by activating stathmin [73]. Specific transfection of miR-142 and miR-223 influences post-transcriptional regulation of proteins in hepatocellular carcinoma (HCCs), which has a suppressive effects on proliferation of hepatocellular carcinoma cells by regulating expressions of stathmin and insulin-like growth factor-1 receptor (IGF-1R) [74] (Fig. 5b).

Stathmin has been identified as a functional target of microRNA-9. Up-regulation of microRNA-9 reduces glioma cell proliferation, migration and vasculogenic mimicry by up-regulating the expression of stathmin [75]. Interestingly, miR-101 expression inhibits the autophagy of hepatocellular carcinoma HepG2 cells by modulating the activity of stathmin, and enhances apoptosis of hepatocellular carcinoma cells by inhibition of autophagy [76]. Another study shows that the expression of miR-101 is negatively correlated with the aggressiveness, growth and angiogenesis in malignant epithelial cancers. Moreover, down-regulation of miR-101 results in the acceleration of cell proliferation and aggressiveness via targeting stathmin, indicating that stathmin is a functional target of miR-101 [77] (Fig. 5c). The clarification of non-coding RNA transcripts is likely conducive to disclose many new mechanisms and pathways for expounding biological phenomena of malignant tumors. Research shows these biological effects of microRNAs on tumors are involved in stathmin signal, influencing the cell cycle control, proliferation, migration and drug resistance. Encouragingly, microRNA molecules are already applying into the clinic as diagnostic and prognostic biomarkers and therapeutic targets and agents.

### Stathmin and chemoresistance of cancers

As shown in Table 2, higher expression of stathmin closely correlates with microtubule-dependent processes and contributes to tumor cell chemoresistance. On the contrary, down-regulation of stathmin significantly reduces the chemoresistance. In this section, studies on relationship between stathmin and chemoresistance of cancers are addressed separately. Recent study demonstrates that overexpression of stathmin influences the efficacy of paclitaxel, suggesting that it can be a negative prognosis indicator for non-small cell lung cancer patients who are treated by both platinum and paclitaxel chemotherapy [11]. NCI-H1299 cells (NSCLC) are evidently resistant to taxol-induced cellular apoptosis and high expression of stathmin is perhaps a crucial determinant of taxol-resistant development in NCI-H1299 cells. Meanwhile, ERK-mediated stathmin is involved in taxol resistance, because blockage of ERK signal improves the sensitivity of tumor cells to taxol [78, 79]. Over-expression of antiapoptotic protein Bcl-2 has been shown to induce chemoresistance. However, blockade of stathmin and Bcl-2 expression can sensitize lung cancer cells to paclitaxel [80]. Dramatically, knockdown of stathmin combined with paclitaxel remarkably promotes the efficacy of inhibiting proliferation of esophageal squamous cell cancer [81], and leads to a significantly higher proportion of cells at G2/M phase, and this antiproliferative effect was accompanied by an increase in apoptosis rates and morphology changes [82].

**Table 2:** The research progress of stathmin and chemoresistance

Publications	Cancer type	Cell lines	Tissues	Anticancer drugs	Notes
Sun [11]	Non-small cell lung cancer	No	Yes	Platinum; paclitaxel	High level of stathmin exhibits poor response to chemotherapy
Lin [78]		Yes	No	Taxol	Inhibition of stathmin expression increases sensitivity to taxol and promotes cellular apoptosis in NCI-H1299 cells
Lin [79]		Yes	No	Taxol	ERK-mediated stathmin is involved in taxol resistance of NCI-H1299 cells; blockage of ERK signal improves sensitivity of tumor cells to taxol
Han [80]		Yes	No	Paclitaxel	Inhibition of stathmin and Bcl-2 expression can sensitize lung cancer cells to paclitaxel
Feng [82]	Esophageal squamous carcinoma	Yes	No	Paclitaxel	Combined chemotherapeutic agent paclitaxel and stathmin siRNA can potentially enhance the therapeutic outcomes of paclitaxel in treating esophageal squamous cell cancer (ESCC)
Wang [16]		Yes	No	Paclitaxel	Silencing of stathmin gene can increase sensitivity of ESCC to paclitaxel and vincristine through G2/M phase block
Zhu [81]		Yes	No	Paclitaxel	Stathmin silencing by siRNA enhances sensitivity of esophageal cancer cells Eca-109 to paclitaxel and induces apoptosis
Balasubramani [83]	Breast cancer	Yes	No	Taxol	Stathmin overexpression protects the cells from taxol-induced abnormal mitoses, and thus induces taxol resistance
Miceli [48]		Yes	No	Taxol	Combination of anti-stathmin therapy and taxol has a more profound inhibition of tumorigenicity
Oda [84]		Yes	No	Zoledronic acid; gefitinib	Down-regulation of stathmin contributes to the effect that combined treatment of Zoledronic acid (Zol) and gefitinib inhibits both invasion and cell proliferation of the bone-seeking clone of breast cancer
Meng [61]	Gastric cancer	Yes	No	Docetaxel	Stathmin siRNA can improve the chemosensitivity of gastric cancer cells to docetaxel and promote apoptosis
Li [24]		Yes	No	Docetaxel	Stathmin mediates docetaxel resistance in transcription factor forkhead box protein M1 (FOXM1)FOXM1-silenced gastric cancer cells
Liu [25]		Yes	No	Docetaxel	Inhibition of stathmin enhances the inhibitory effects of docetaxel on the proliferation of gastric cancer cells
Werner [85]	Endometrial carcinoma	Yes	Yes	Paclitaxel	Knock-down of stathmin improves sensitivity to paclitaxel in endometrial carcinoma cells
Wosnitzer [35]	Bladder cancer	No	Yes	Taxane	Bladder cancer those who have tumors with low tau/stathmin protein expression show a better response to taxane
Mitra [86]	Retinoblastoma	Yes	Yes	Paclitaxel	Inhibition of stathmin enhances the cytotoxic effect of paclitaxel
Song [63]	Glioma	Yes	No	Temozolomide	Stathmin silencing inhibits invasion and enhances chemotherapy sensitivity of stem cells derived from glioma cells
Feng [87]	Osteosarcoma	Yes	No	Arsenic trioxide; doxorubicin	Down-regulation of stathmin significantly enhances reversion of ADM resistance in MG63/dox by As2O3
Wu [88]	Colorectal cancer	Yes	No	5-fluorouracil	Silencing of stathmin significantly improves chemoresponse to the classical colorectal cancer therapeutic agent, 5-FU
Watanabe [18]	Extrahepatic cholangiocarcinoma	Yes	Yes	Paclitaxel	Silencing of stathmin inhibits proliferation and increases sensitivity of extrahepatic cholangiocarcinoma cells to paclitaxel

*NCI-H1299* lung adenocarcinoma cell lines, *ERK* extracellular regulated protein kinases, *Bcl-2* B-cell lymphoma-2, *ESCC* esophageal squamous cell cancer, *shRNA* short hairpin RNA, *siRNA* small interfering RNA, *FOXM1* transcription factor forkhead box protein M1, *ADM* doxorubicin, *As*_*2*_ *O*_*3*_ arsenic trioxide, *5-FU* 5-fluorouracil

After treated by paclitaxel or vincristine, esophageal squamous cell carcinoma (ESCC) cells of stathmin silencing are more likely to enter G2 but less likely to enter mitosis than control cells, suggesting that silencing of stathmin gene increases sensitivity of ESCC to paclitaxel and vincristine through G2/M phase block [16]. Overexpression of stathmin reduces microtubule dynamicity of cells and sensitivity to taxol, which mainly because overexpression of stathmin protects the cells from taxol-induced abnormal mitoses to lead to taxol resistance [83]. However, combination of anti-stathmin therapy and taxol had a more profound inhibition of tumorigenicity, as both agents target the microtubule pathway [48]. In addition, combined treatment of zoledronic acid and gefitinib synergistically inhibits both invasion and cell proliferation of the bone-seeking clone, but not those of the breast cancer MDA-MB-231 cells. Down-regulation of stathmin of these cooperative effects suggests that it may be a promising target molecule for blocking bone metastasis of breast cancer [84].

Research shows that stathmin silencing recovers the chemosensitivity of gastric cancer cells to docetaxel, arrests cells at the sub-G1 stage, induces apoptosis and inhibits the growth of transplantation tumor [61]. Overexpression of transcription factor forkhead box protein M1 (FOXM1) mediates resistance to docetaxel-induced apoptosis in gastric cancers, and stathmin correlates with resistance to docetaxel in FOXM1-silenced gastric cancer cells, indicating that stathmin is effective downstream signal of FOXM1 [24]. Moreover, depletion of stathmin by antisense oligodeoxynucleotide promotes the antitumor effects of docetaxel to gastric cancer cells, and combination treatment of stathmin inhibition and docetaxel shows a synergistic effect [25]. A protein-binding assay reveals that p27 can be bounded to stathmin of cytoplasm in extrahepatic cholangiocarcinoma (EHCC) cells; moreover, down-regulation of stathmin leads to accumulation of p27, which suppresses proliferation and promotes sensitivity of EHCC cells to paclitaxel [18]. Knock-down of stathmin enhances sensitivity to paclitaxel in endometrial carcinoma cells [85] and also enhances the cytotoxic effect of paclitaxel to retinoblastoma [86]. Inspiringly, in bacillus Calmette–Guérin refractory bladder cancer, patients who have tumors with low stathmin expression seem to have a better response to taxane therapy [35].

Glioma stem cells (GSCs) are usually resistant to chemotherapy and radiotherapy, but silencing of stathmin can improve the sensitivity of glioma stem cells to temozolomide [63]. Arsenic trioxide (As_2_O_3_) and doxorubicin (ADM) combination treatment markedly inhibits cell proliferation of ADM-resistant MG63 (MG63/dox) osteosarcoma cells, and induces apoptosis of MG63/dox cells. Surprisingly, down-regulation of stathmin significantly enhances the reversion of ADM resistance in MG63/dox by As_2_O_3_, and As_2_O_3_ also reverse ADM resistance in MG63/dox cells by down-regulation of stathmin [87]. In addition, 5-FU chemoresponse to the classical colorectal cancer can be improved by silencing of stathmin via a caspase-6 (CASP6)-dependent signal. Interestingly, the function of stathmin is independent of p53 but requires phosphorylations at S25 or S38 [88].

From the above, we can say that assessment of stathmin expression should be considered to use for selection of patients before chemotherapy of some drugs. In addition, research on the relationship between chemoresistance and stathmin should be reinforced, which will be useful to identify a potential chemoresistance marker and to develop a new molecular targeted drug.

### Stathmin-dependent molecular targeting therapy based on interfering with stathmin function

In the last 5 years, many investigations have suggested that stathmin is a potential target for treatment of solid malignant tumors. Especially, a variety of target-specific anti-stathmin effectors, including ribozymes, monoclonal antibody, shRNA and siRNA have been used extensively to decrease expression of stathmin in vitro and vivo to investigate the therapeutic strategies targeted towards stathmin.

As shown in Table 3, these studies point out that down-regulation of stathmin significantly reduces cell proliferation, clonal growth, cell motility and metastasis, and increase apoptosis of malignant tumors. For instance, knockdown of stathmin significantly reduces pancreatic cancer cell viability, colony formation, and even retards pancreatic tumor growth in nude mice [27].Although leukemia is not a solid tumor, stathmin silencing still reduces cell proliferation and clonogenicity of U937 and Namalwa leukemia cells [66]. More widely, siRNA-mediated silencing of stathmin has been shown to suppress the proliferation, invasion and metastasis of nasopharyngeal carcinoma (NPC) cells [60], hepatoma [4], retinoblastoma [86], endometrial carcinoma [33], bladder cancer [36] and glioma [89], and significantly induces the apoptosis of tumor cells [54, 60, 62]. Adenovirus-mediated gene transfer of anti-stathmin ribozyme inhibits cell proliferation and clonogenicity in both ER-positive and ER-negative breast cancer cells [48] and knockdown of stathmin can attenuate the miR-101-mediated enhancement of cell growth and metastasis [77]. Stathmin promoter-driving Aurora A shRNA adenoviral system may has potential use, which is considered as adjuvant tumor-specific therapy method, in the treatment of human breast carcinomas [55]. To lung cancer cells, knockdown of stathmin results in a remarkable decrease in cellular proliferation and invasion [3], and monoclonal antibodies against stathmin also inhibit the proliferation of human lung carcinoma QG-56 cells, and even result in a significantly higher apoptosis rate [54]. Moreover, knockdown of stathmin impairs cell proliferation and migration of esophageal squamous cells [13, 14, 16], and leads to cell cycle arrest in G2/M phase [16].

**Table 3:** Summary of stathmin targeted treatment against human tumors

Publication	Cancer type	Molecule and mechanism	Activity	Notes
Lu [27]	Pancreatic Cancer	Inhibitors of stathmin expression	mRNA downregulation	Knockdown of stathmin significantly reduces pancreatic cancer cell viability, colony formation. Furthermore, silence of stathmin retards pancreatic tumor growth in nude mice
Machado-Neto [29]	Leukemia	siRNA	mRNA downregulation	Stathmin silencing in U937 and Namalwa leukemia cells reduces cell proliferation and clonogenicity
Wu [60]	Nasopharyngeal carcinoma	siRNA	mRNA downregulation	The siRNA-mediated silencing of stathmin suppresses proliferation, invasion and metastasis, and induces apoptosis of nasopharyngeal carcinoma (NPC) cells
Miceli [48]	Breast cancer	Ribozyme	mRNA downregulation	Adenovirus-mediated gene transfer of anti-stathmin ribozyme inhibits proliferation and clonogenicity in both ER-positive and ER-negative breast cancer cells
Wang [77]		siRNA	mRNA downregulation	Knockdown of stathmin attenuates down-regulation of miR-101-mediated enhancement of cell growth and metastasis
Long [55]		shRNA	mRNA downregulation	Stathmin promoter-driving Aurora A shRNA adenoviral system has a potential use, which acts as adjuvant tumor-specific therapy method, in treatment of human breast carcinomas
Nie [3]	Lung cancer	siRNA	mRNA downregulation	Knockdown of stathmin in lung cancer cells results in a decrease in cellular proliferation and invasion
Yuan [54]		Monoclonal antibodies	Protein downregulation	Monoclonal antibodies against stathmin inhibit proliferation of human lung carcinoma QG-56 cells and result in a significantly higher apoptosis rate
Hsieh [4]	Hepatoma	siRNA	mRNA downregulation	Silencing of stathmin expression via RNA interference suppresses invasion activity, while ectopic expression of stathmin enhances cell invasion and caused polyploidy of cells
Wang [16]	Esophageal squamous cell carcinoma	shRNA-transfected	mRNA downregulation	Flow cytometry and mitotic index assays show that knockdown of stathmin in esophageal squamous cell carcinoma Eca109 and TE-1 cells leads leads to cell cycle arrest in G2/M phase
Liu [13]		siRNA	mRNA downregulation	Knockdown of stathmin with siRNA impairs cell migration in esophageal squamous cell carcinoma KYSE30 and KYSE410 cells
Wang [14]		shRNA plasmid	mRNA downregulation	Down-regulation of stathmin significantly inhibits cell proliferation, cell migration in vitro, and tumorigenicity in vivo
Machado-Neto [62]	Myeloproliferative neoplasms	siRNA	mRNA downregulation	Silencing of stathmin significantly reduces cell proliferation and clonal growth, and increases apoptosis induced by ruxolitinib
Mitra [86]	Retinoblastoma	Short interfering RNA	mRNA downregulation	Short interfering RNA-mediated transient stathmin down-regulation results in a marked inhibition of retinoblastoma cell proliferation and cell invasion in vitro
He [33]	Endometrial carcinoma	siRNA	mRNA downregulation	Knockdown of stathmin inhibits endometrial carcinoma cell aggressive behaviors.
Liu [25]	Gastric cancer	Antisense oligodeoxynucleotide	mRNA downregulation	Stathmin transfected by antisense oligodeoxynucleotide significantly inhibits proliferation of gastric cancer SGC 7901 cells
Akhtar [91]		shRNA	mRNA downregulation	Stathmin shRNA-treated tumors are significantly regressed as compared with that of the tumor injected with non-silencing shRNA, proposing a potential use of local injection of lentivirus-delivered shRNA for the treatment of early localized human gastric carcinoma
Akhtar [90]		siRNA	mRNA downregulation	Lentiviral-mediated RNA interference targeting stathmin gene in human gastric cancer cells inhibits proliferation in vitro and tumor growth in vivo
Hemdan [36]	Bladder cancer	siRNA	mRNA downregulation	Growth and migration of urinary bladder cancer cell line T24 cells are significantly reduced by stathmin-siRNA
Dong [89]	Glioma	siRNA	mRNA downregulation	When down-regulation of stathmin, cell viability of glioma is reduced, apoptosis rate increases and migration of vascular endothelial cells is suppressed significantly

*mRNA*, messenger RNA, *U937* histiocytic lymphoma cells was established by Dr. K. Nilsson’s laboratory in 1974, *shRNA* short hairpin RNA, *siRNA* small interfering RNA, *NPC* nasopharyngeal carcinoma, *ER* estrogen receptor, *miR* small non-protein-coding regulatory RNAs, *Aurora A* Aurora kinase A, *QG-56* human lung carcinoma QG-56 cells, *Eca109 and TE-1 cells* esophageal squamous cell carcinoma Eca109 and TE-1 cells, *KYSE30 and KYSE410* esophageal squamous cell carcinoma KYSE30 and KYSE410 cells, *SGC 7901* gastric cancer SGC 7901 cells, *T24* bladder cancer cell line T24 cells

The depletion of stathmin by antisense oligodeoxynucleotide significantly inhibits the proliferation of gastric cancer cells [25, 90]. Excitedly, the local injection of stathmin lentivirus-delivered shRNA could be used to treat early localized human gastric carcinoma [91]. Bifunctional small hairpin RNAs (bi-shRNAs) is functional miRNA/siRNA composite; one study shows that a single intratumoral injection of pbi-sh-stathmin reduces growth of tumor xenograft derived from colorectal cancer CCL-247 cells, and also significantly inhibits the growth of tumorgrafts derived from primary melanoma and osteosarcoma xenograft [92]. In human cancers, stathmin is usually overexpressed and anti-stathmin treatment usually reduces cell proliferation, clonal growth, cell motility, metastasis and increases apoptosis. So, an‘anti-stathmin’ targeted therapy could be a potential strategy to cure malignant tumors.

### Perspective and limitation

Up to now, we still know relatively little about how the stathmin regulates tumor proliferation, motility, migration and occurence of metastasis. However, the abnormal expression of stathmin in tumor cells has provided to be a feasible approach for the development of stathmin-dependent molecular targeting therapy. This way is also fraught with new challenges, for instance, efficient molecules and compounds able to specifically reduce stathmin expression and decrease stathmin activity have not been developed, yet. The clinical application of stathmin-dependent molecular targeting therapy is lagging way behind, which needs further hard study to explore and discover. In addition, combination treatment of anti-stathmin and other chemotherapy drugs still needs to further study. Anyway, elucidating the function of the stathmin in malignant tumors will effectively disclose the mechanisms of tumor progress and metastasis as well as greatly promote the development of new anticancer therapies.

## Conclusion

Stathmin expression has been found to be increased in a variety of cancers and high expression of stathmin can potentially promote cell proliferation, motility and metastasis of malignant tumors. However, many target-specific anti-stathmin investigations have been demonstrated to reduce cell proliferation, clonal growth, cell motility and metastasis, and to increase apoptosis of malignant tumors. Whether or not stathmin proves to be a significant therapeutic target, the identification of specific target, development of effective therapeutic drugs and construction of drug delivery vehicles, will all bring a new challenge. Hopefully, this understanding predicts that stathmin-dependent molecular targeting therapy for malignant tumor will soon come out.

## Abbreviations

ADM: doxorubicin
AGS: gastric adenocarcinomas cell lines
Akt: v-akt murine thymoma viral oncogene
ANKHD1: Ankyrin Repeat and KH Domain Containing 1 protein
As2O3: arsenic trioxide
Bcl-2: B-cell lymphoma-2
BE(2)-C: neuroblastoma cell lines
bi-shRNAs: bifunctional small hairpin RNAs
CCL-247: human colorectal cancer cells
CASP6: caspase-6
CDC2: cyclin-dependent kinase 1
CDK: cyclin dependent kinase
CDK5: cyclin-dependent kinase-5
CIN3: cervical intraepithelial neoplasias 3
CITs: indoly-chalcones
CREB1: leucine zipper transcription factor
DNA: deoxyribonucleic acid
DNA-PKCS: catalytic subunit of the DNA-dependent protein kinase
2-DE: two-dimensional electrophoresis
2-D DIGE: two dimension difference gel electrophoresis
E1: colorectal cancer cell lines
E2F1: transcription factor 1
EC: esophageal carcinoma
Eca109: esophageal squamous cell carcinoma cell lines
EC9706: esophageal squamous cell carcinoma cell lines
EHCC: extrahepatic cholangiocarcinoma
ELISA: enzyme-linked immunosorbent assay
EOC: epithelial ovarian carcinomas
ESCC: esophageal squamous cell carcinoma
EF1α: EF1α promoter
E2F1: E2F transcription factor 1
EMT: epithelial-mesenchymal transition
ER: estrogen receptor
ERK: extracellular regulated protein kinases
5-FU: 5-fluorouracil
FISH: fluorescence in situ hybridization
FCM: low cytometry
FOXM1: transcription factor forkhead box protein M1
FTE: fallopian tube epithelium
GSCs: glioma stem cells
HCC: hepatocellular carcinoma
HCT-116: colorectal cancer cell lines
HepG2: hepatocellular carcinoma cell line
HIF-1α: hypoxia-inducible factor-1
HSP90: Heat shock protein 90
IGF-1R: insulin-like growth factor-1 receptor
IHC: immunohistochemistry
ISH: in situ hybridization
JAK: Janus Kinase
JNK: c-JunN-terminalkinase
